# Staphylococcus epidermidis MSCRAMM SesJ Is Encoded in Composite Islands

**DOI:** 10.1128/mBio.02911-19

**Published:** 2020-02-18

**Authors:** Srishtee Arora, Xiqi Li, Andrew Hillhouse, Kranti Konganti, Sara V. Little, Sara D. Lawhon, David Threadgill, Samuel Shelburne, Magnus Hook

**Affiliations:** a Center for Infectious and Inflammatory Diseases, Institute of Biosciences and Technology, Texas A&M University Health Science Center, Houston, Texas, USA; b Department of Infectious Diseases, Division of Internal Medicine, The University of Texas MD Anderson Cancer Center, Houston, Texas, USA; c Institute for Genome Sciences and Society, Texas A&M University, College Station, Texas, USA; d Veterinary Medicine and Biomedical Sciences, Texas A&M University, College Station, Texas, USA; Skirball Institute of Biomolecular Medicine, New York University Medical Center

**Keywords:** ACME, cell wall-anchored proteins, MSCRAMM, SCC*mec*, SesJ

## Abstract

Staphylococcus epidermidis is a leading cause of nosocomial infections in patients with a compromised immune system and/or an implanted medical device. Seventy to 90% of S. epidermidis clinical isolates are methicillin resistant and carry the *mecA* gene, present in a mobile genetic element (MGE) called the staphylococcal cassette chromosome *mec* (SCC*mec*) element. Along with the presence of antibiotic and heavy metal resistance genes, MGEs can also contain genes encoding secreted or cell wall-anchored virulence factors. In our earlier studies of S. epidermidis clinical isolates, we discovered S. epidermidis surface protein J (SesJ), a prototype of a recently discovered subfamily of the microbial surface component recognizing adhesive matrix molecule (MSCRAMM) group. MSCRAMMs are major virulence factors of pathogenic Gram-positive bacteria. Here, we report that the *sesJ* gene is always accompanied by two glycosyltransferase genes, *gtfA* and *gtfB*, and is present in two MGEs, called the arginine catabolic mobile element (ACME) and the staphylococcal cassette chromosome (SCC) element. The presence of the *sesJ* gene was associated with the left-hand direct repeat DR_B or DR_E. When inserted via DR_E, the *sesJ* gene was encoded in the SCC element. When inserted via DR_B, the *sesJ* gene was accompanied by the genes for the type 1 restriction modification system and was encoded in the ACME. Additionally, the SCC element and ACME carry different isoforms of the SesJ protein. To date, the genes encoding MSCRAMMs have been seen to be located in the bacterial core genome. Here, we report the presence of an MSCRAMM in an MGE in S. epidermidis clinical isolates.

## Abstract (precis)

S. epidermidis is an opportunistic bacterium that has established itself as a successful nosocomial pathogen. The modern era of novel therapeutics and medical devices has extended the longevity of human life, but at the same time, we also witness the evolution of pathogens to adapt to newly available niches in the host. Increasing antibiotic resistance among pathogens provides an example of such pathogen adaptation. With limited opportunities to modify the core genome, most of the adaptation occurs by acquiring new genes, such as virulence factors and antibiotic resistance determinants present in MGEs. In this study, we describe that the *sesJ* gene, encoding a recently discovered cell wall-anchored protein in S. epidermidis, is present in both ACME and the SCC element. The presence of virulence factors in MGEs can influence the virulence potential of a specific strain. Therefore, it is critical to study the virulence factors found in MGEs in emerging pathogenic bacteria or strains to understand the mechanisms used by these bacteria to cause infections.

## INTRODUCTION

Staphylococcus epidermidis is an opportunistic pathogen associated with infections in patients with an implanted foreign body and/or with a compromised immune response (1–3). It is also part of the normal human skin microbiome. Historically, S. epidermidis was not known as a powerful pathogen. However, advances in medicine, particularly the use of implants and immune-suppressant drugs, have provided opportunities for S. epidermidis to expand its habitat. In addition, the continuous overuse of antibiotics has led to a dramatic increase in the occurrence of multiresistant strains of many pathogens, including S. epidermidis. Now, the importance of S. epidermidis as a modern-day pathogen is growing, as it has established itself as a successful nosocomial pathogen.

Recent studies to identify the genetic background of infectious S. epidermidis isolates around the globe revealed that a few sequence types (STs) are primarily associated with serious infection. ST2, the founder sequence type of clonal complex 2 (CC2), is the predominant and geographically most widespread clone of invasive S. epidermidis (4–6). A high frequency of genetic recombination within CC2 has resulted in a large number of sequence types in this genetic lineage (5–7). S. epidermidis has also been successful as an opportunistic pathogen perhaps partly because of its capability of high-frequency genetic recombination and gene acquisition.

Bacteria can adapt to a selective environmental pressure by acquiring mobile genetic elements (MGEs) encoding specific genes, such as novel virulence factors and antibiotic resistance determinants. Seventy to 90% of S. epidermidis clinical isolates are methicillin resistant and carry the *mecA* gene in the MGE called the staphylococcal cassette chromosome *mec* (SCC*mec*) element (6, 7). The SCC*mec* elements have four characteristic features: (i) the presence of the methicillin resistance gene *mecA*; (ii) the presence of the cassette chromosome recombinase genes *ccrAB* and/or *ccrC*, which mediate insertion and excision of the cassette; (iii) an insertion at the integration site sequence *att* in the *orfX* gene (also known as the *rlmH* gene); and (iv) the presence of the flanking direct repeats (DRs) on both sides of the integrated SCC*mec* (8). The *mecA* gene complex and *ccr* gene complex are connected to each other and to the ends of the cassette by joining regions (J1, J2, and J3). To date, 13 SCC*mec* elements have been identified in staphylococci, based on different combinations of the *mec* gene complex and the *ccr* gene complex (9–11). Nine of these SCC*mec* elements are found in human isolates (10, 11).

Genetic recombination has led to multiple novel configurations of the staphylococcal cassette chromosome (SCC) elements deviating from the identified types and subtypes. Pseudo elements missing either the recombinase genes (ΨSCC*mec*) or a functional *mec* gene complex have been described in the literature (8, 12–14). The SCC non-*mec* elements contain other genes that benefit the organism instead of the *mecA* gene. For example, SCC*cap1* contains the genes for capsular polysaccharide, while SCC*Hg* carries the mercury resistance operon (15, 16). To avoid confusion among the growing list of SCC*mec* types and subtypes, the International Working Group on the Classification of Staphylococcal Cassette Chromosome Elements has set up guidelines to name SCC elements (8).

In addition to the SCC elements, the arginine catabolic mobile element (ACME) has frequently been observed in coagulase-negative staphylococci, especially S. epidermidis. About 50 percent of clinical isolates of S. epidermidis tested had ACME (17, 18). An ACME island which enhances the pathogenic fitness, i.e., which enhances colonization and transmission, was found in Staphylococcus aureus USA 300 strains responsible for an epidemic in the United States (19, 20). ACME is often found together with SCC*mec* and can be inserted either downstream or upstream of SCC*mec*, thus forming a composite island (CI) (21, 22). When found upstream of SCC*mec*, ACME is inserted at the *orfX* gene. The *arc* operon for the arginine deiminase pathway and the *opp* genes for the oligopeptide permease operon are the two main gene clusters present in ACME (21, 23). There are three well-recognized allotypes of ACME: ACME I contains both the *opp* and *arc* clusters, ACME II contains the *arc* gene cluster, and ACME III contains the *opp* gene cluster (18). However, recently, ACME IV, containing the *arc* and *kdp* operon, and ACME V, containing the *arc*, *opp*, and *kdp* operon, were described (24). The *kdp* operon encodes a high-affinity potassium uptake system.

Along with the presence of antibiotic and heavy metal resistance genes, MGEs can also contain genes encoding secreted or cell wall-anchored (CWA) virulence factors. One such virulence factor, plasmin-sensitive protein (Pls), a CWA protein from S. aureus, is present in the SCC*mec* type I J region (25). Pls plays a role in biofilm formation through the G5 repeats as well as the glycosylated serine aspartate dipeptide repeat region (26). In a mouse septic arthritis model, the presence of the *pls* gene also led to more frequent joint infection and severe arthritis (27).

CWA proteins can be grouped by either their structural or their functional similarities. One of the groups of structurally related CWA proteins is the microbial surface component recognizing adhesive matrix molecules (MSCRAMMs). MSCRAMMs are known virulence factors in Gram-positive pathogens. Recently, we reported on a novel subfamily of MSCRAMM proteins characterized by the N-terminal repeat region. Members of this subfamily of MSCRAMMs are present in multiple coagulase-negative staphylococci. SesJ is the prototype of this newly discovered N-terminal repeat-containing subfamily of MSCRAMM (28). Here, we report the presence of the *sesJ* gene in ACME and SCC*mec* elements in S. epidermidis.

## RESULTS

### Epidemiology study.

We analyzed 171 S. epidermidis strains isolated from the blood of patients that presented with symptoms of bloodstream infections at the MD Anderson Cancer Center in Houston, TX. As reported earlier, all the isolates were sequenced using an Illumina MiSeq system (29). Using an *in silico* approach, we determined the prevalence of the *sesJ* gene in these isolates. The *sesJ* gene was present in 30/171 (18%) isolates (Table 1). Among the different STs, the *sesJ* gene was detected in 6/34 (18%) ST2 isolates, 16/58 (26%) ST5 isolates, and 6/6 (100%) ST210 isolates. Two of 16 singleton STs were also positive for the *sesJ* gene (Table 1). In addition, we divided the collection of strains into methicillin-resistant S. epidermidis (MRSE) and methicillin-susceptible S. epidermidis (MSSE) isolates. This study included 139 (81%) MRSE isolates and 32 (19%) MSSE isolates. The *sesJ* gene was present in 26/139 (19%) MRSE isolates and 4/32 (13%) MSSE isolates (Table 2). Out of the 4 *sesJ*-positive (*sesJ*^+^) MSSE isolates, 2 isolates belonged to ST210 and the other 2 isolates were singletons (ST218 and ST57). Although the *sesJ* gene was present in both MRSE and MSSE isolates, the data presented above points toward the presence of the *sesJ* gene in specific STs. Furthermore, it is noteworthy that all ST210 isolates in our collection were *sesJ*^+^.

**TABLE 1:** Prevalence of *sesJ* gene

ST	No. of isolates	No. (%) of *sesJ*^+^ isolates
ST2	34	6 (18)
ST83	17	0
ST210	6	6 (100)
ST5	58	16 (27)
ST16	5	0
ST20	3	0
ST22	5	0
ST59	4	0
ST69	3	0
ST130	3	0
ST6	4	0
Rare	29	2
		
Total	171	30 (17)

**TABLE 2:** Presence of *sesJ* gene in MRSE and MSSE isolates

Isolate	No. of isolates	No. (%) of *sesJ*^+^ isolates
MRSE	139	26 (19)
MSSE	32	4 (13)

### The *sesJ* gene is present in a CI inserted in the *orfX* gene.

**FIG 1:**
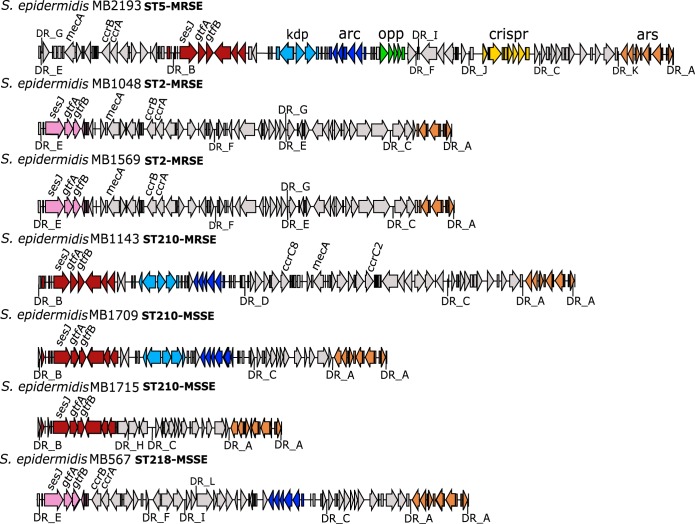
Schematic representation of the *sesJ* gene in MGEs containing SCC elements, ACME, and the *ars* operon. Each ORF is represented by an arrow, with the direction of the arrow indicating the direction of transcription. Each group of genes is represented by a different color, as follows: red, the set of genes, including *sesJ*, associated with DR_B; pink, the set of genes, including *sesJ*, associated with DR_E; light blue, the *kdp* operon; dark blue, the *arc* operon; green, the *opp* operon; yellow, the *crispr* operon; and brown, the *ars* operon. The DRs are named, and the black line pointing to the DR name represents the location of the DR. ST and methicillin resistance status are mentioned in bold next to the isolate name.

The presence of the *sesJ* gene in a relatively small portion (18%) of the isolates indicated that, unlike other MSCRAMM genes encoded in the core genome, the *sesJ* gene might be present in an MGE. MGEs are difficult to assemble using the short Illumina reads alone. Therefore, we selected 7 *sesJ*^+^ isolates from different STs, including MRSE and MSSE isolates, for long-read sequencing. Whole-genome sequencing revealed that the *sesJ* gene in both the MRSE and the MSSE isolates was present in MGEs inserted in the *orfX* gene (Fig. 1). Further analysis revealed that the *sesJ* gene was present in two well-known MGEs: ACME and SCC elements. Each *sesJ*^+^ isolate contained only one copy of the *sesJ* gene encoded in either an ACME or an SCC element. In ST2 MRSE isolates MB1048 and MB1569, the *sesJ* gene was present in the J3 region of SCC*mec* type IV. Along with the ST2 isolates, the *sesJ* gene was also encoded in an SCC element present in MSSE ST218 isolate MB567. On the other hand, the *sesJ* gene was present in ACME IVa in ST210 isolates (MB1143 and MB1709) and in ACME V in ST5 isolate MB2193.

All the MGEs containing the *sesJ* gene also contained the *ars* operon (Fig. 1). The *ars* operon confers resistance to arsenite, arsenate, and antimonite (30). In the MRSE and MSSE isolates investigated, the MGE containing the *sesJ* gene was demarcated by either DR_E or DR_B at the left junction of the element. Further investigation revealed that the SCC element containing the *sesJ* gene was demarcated by DR_E at the left junction and that the ACME containing the *sesJ* gene was demarcated by DR_B at the left junction (Fig. 1 and Table 3).

**TABLE 3:** Sequences and names of direct repeats

Direct repeat name	Sequence
DR_A	GAAGCGTATCGTAAGTGA
DR_B	GAAAGTTATCATAAGTGA
DR_C	GAAGCGTATAATAAGTAA
DR_D	GAAGCGTATCATAAATAA
DR_E	GAAGCATATCATAAATGA
DR_F	GAAGCGTATCACAAATAA
DR_G	GAAGCTTATCATAAGTAA
DR_H	GAATCATATCAAAAATGT
DR_I	GAAGCATATCATAAGTGA
DR_J	GAAGGGTATCGTAAGTGA
DR_K	GAAGCGTATCGTAAGTAA
DR_L	GAAGCGTATCATAAGTGA

### Organization of the SCC element containing the *sesJ* gene.

**FIG 2:**
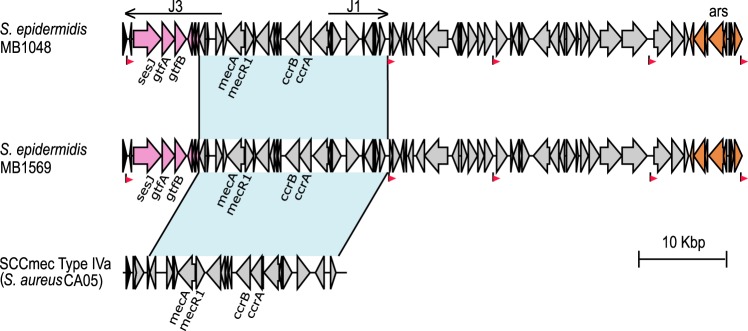
Schematic representation of the MGEs containing the *sesJ* gene in S. epidermidis MB1048 and MB1569. The previously described SCC*mec* type IVa from S. aureus CA05 is included for comparison. The region in light blue with a black border represents a region of at least 99% identity. Each ORF is represented by an arrow, with the direction of the arrow indicating the direction of transcription. Each group of genes is represented by a different color, as follows: pink, the set of genes, including *sesJ*, associated with DR_E, and brown, the *ars* operon. DRs are indicated as red flags pointing in the same direction as the DR, and the black line associated with the red flag represents the location of the DR.

The two ST2 MRSE isolates MB1048 and MB1569 contained SCC*mec* type IV and harbored the *sesJ* gene in the J3 region joining the *orfX* gene and the *mec* complex (Fig. 2). The SCC*mec* element in both MB1048 and MB1569 contained *mec* complex B and *ccr* complex 2 (confirmed with primers 2F/2R and 3F/3R, respectively; see Fig. S1a in the supplemental material) and was flanked by DR_E and DR_F (Fig. 1). In both isolates, the SCC*mec* element containing the *sesJ* gene was inserted in the *orfX* gene (confirmed with primer 1F, located in the *mecA* gene, and primer 14R, located in the *sesJ* gene; Fig. S1a), creating DR_E. Compared with the sequence of SCC*mec* type IVa from S. aureus CA05, the SCC*mec* elements in MB1048 and MB1569 shared 99% nucleotide sequence identity in the region shown in light blue in Fig. 2. Additionally, the *ccrB2* and *ccrA2* genes from MB1048 and MB1569 had 96% nucleotide sequence identity with the *ccr* genes from S. aureus CA05. However, the SCC*mec* element from MB1048 and MB1569 had a J3 region different from that in SCC*mec* type IVa. The J3 region of SCC*mec* type IV from MB1048 and MB1569 contained eight open reading frames (ORFs), including the *sesJ* gene and the two glycosyltransferase genes *gtfA* and *gtfB*, encoded by a region 88 bp downstream of the *sesJ* gene. The presence of the two glycosyltransferase genes *gtfA* and *gtfB* downstream of the *sesJ* gene was confirmed with primer 5F, located in the *sesJ* gene, and primer 5R, located in the *gtfB* gene (Fig. S1a). The remaining five ORFs in the J3 region encoded hypothetical proteins. No direct repeat was identified between DR_E and DR_F flanking the SCC*mec* elements. The region downstream of the SCC*mec* element between DR_F and DR_C shares identity with the forward flank novel content found in the ST2 S. epidermidis BPH0662 isolate. Downstream of the novel content was the *ars* operon (confirmed with primers 16F/16R; Fig. S1a).

In ST218 MSSE isolate MB567, the CI containing the *sesJ* gene was inserted into the *orfX* gene (confirmed with primers 1F/14R; Fig. S1b). Like ST2 MRSE isolates MB1048 and MB1569, the left junction in MB567 was also marked by DR_E (Fig. 1). The region between DR_E and DR_F met the criteria for an SCC element; i.e., it was inserted into the *orfX* gene, contained the *ccr* genes, and was flanked by direct repeats (Fig. 1). Per the guidelines set forth by the International Working Group on the Classification of Staphylococcal Cassette Chromosome Elements, we have named the region between DR_E and DR_F in S. epidermidis MB567 SCC*sesJ*. SCC*sesJ* contains eight ORFs from the J3 region of SCC*mec* type IV present in MB1048 and MB1569 and the *ccrB2* and *ccrA2* genes (confirmed with primers 15F/21R; Fig. S1b) and another six ORFs encoding hypothetical proteins downstream of the *ccr* genes (Fig. 1). Altogether, the CI of strain MB567 (CI_MB567_) contains SCC*sesJ*; ΔACME II, demarcated by DR_M and DR_A (confirmed with primer pairs 22F/8R-2 and 24F/24R; Fig. S1b); and the *ars* operon (confirmed with primers 13F-2/13R-2; Fig. S1b).

### Organization of ACME containing the *sesJ* gene.

**FIG 3:**
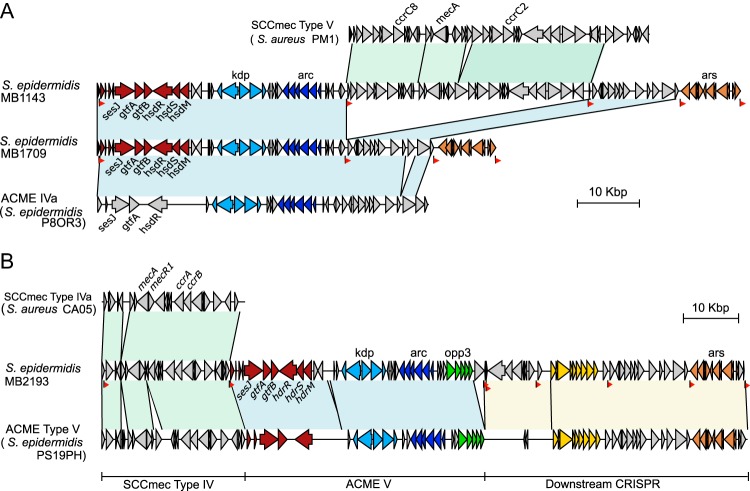
Schematic representation of the ACME containing the *sesJ* gene. The region in light blue with a black border represents a region of at least 99% identity with the sequence of the ACME. The region in green with a black border represents a region of 97% identity with the sequence of SCC*mec*. Each ORF is represented by an arrow, with the direction of arrow indicating the direction of transcription. Each group of genes is represented by a different color, as follows: red, the set of genes, including *sesJ*, associated with DR_B; pink, the set of genes, including *sesJ*, associated with DR_E; light blue, the *kdp* operon; dark blue, the *arc* operon; green, the *opp* operon; yellow, the *crispr* operon; and brown, the *ars* operon. DRs are indicated as red flags pointing in the same direction as the DR, and the black line associated with the red flag represents the location of the DR. (A) Comparison of the MGEs in S. epidermidis MB1143 and MB1709 with the previously described ACME IVa from S. epidermidis P8OR3 and SCC*mec* type V from S. aureus PM1. (B) Comparison of the MGEs in S. epidermidis MB2193 with the previously described ACME V from S. epidermidis PS19PH and SCC*mec* type IVa from S. aureus CA05.

Next, we analyzed the CIs in isolates MB1143 (ST210 MRSE), MB1709 (ST210 MSSE), MB1715 (ST210 MSSE), and MB2193 (ST5 MRSE) containing the *sesJ* gene and demarcated by DR_B at the left junction (Fig. 1). The number of subtypes of ACME was recently expanded from three to five. Per the new classification, ACME IV contains the *arc* operon and the *kdp* operon and ACME V contains the *arc* operon, the *opp* operon, as well as the *kdp* operon (24). In S. epidermidis isolates MB1709 (MSSE) and MB1143 (MRSE), the *sesJ* gene is present in ACME IVa (Fig. 3A). ACME IVa in both S. epidermidis MB1709 and MB1143 contains the *kdp* operon and the *arc* operon and is inserted into the *orfX* gene (confirmed with primer pairs 8F/8R and 1F/14R; Fig. S1c and d, respectively). Previously reported ACME IVa from S. epidermidis P8OR3 (ST210) shares 99% sequence identity with the ACME IVa containing the *sesJ* gene in S. epidermidis MB1709 (Fig. 3A). The differences in the two ACME IVa subtypes are located toward the 3′ end. ACME IVa in S. epidermidis MB1709 has an additional ORF not encoded in the S. epidermidis P8OR3 and lacks the last two ORFs from ACME IVa in S. epidermidis P8OR3. Downstream of the ACME IVa in S. epidermidis MB1709 is the *ars* operon, demarcated by the DR_A repeats (confirmed with primers 13F/13R; Fig. S1c). Similar to S. epidermidis P8OR3, no direct repeats were identified between DR_B and DR_C.

Next, methicillin-resistant S. epidermidis strain MB1143 (ST210) was found to harbor SCC*mec* type V inserted in ACME IVa (confirmed with primers 17F/18R and 19F/17R; Fig. 3A and Fig. S1d). SCC*mec* type V was inserted into the DR_C present in ACME IVa, resulting in demarcation by DR_D and DR_C. In S. epidermidis MB1143, the CI containing ACME IVa and SCC*mec* type V was inserted into the *orfX* gene (confirmed with primers 1F/14R; Fig. S1d). The presence of the *kdp* and *arc* operons in ACME IVa was confirmed with primers 8F/8R (Fig. S1d). Similarly, the presence of the *ccrC8* and *ccrC2* genes, and *mec* complex C2 was confirmed with primer pairs ccrc8F/ccrc8R, ccrc2F/ccrc2R, and 2F/25R, respectively (Fig. S1d). A comparison of the CI present in MB1143 with ACME IVa and SCC*mec* type V revealed that the *sesJ* gene was present in ACME IVa upstream of the *kdp* operon and the *arc* operon. The regions of ACME IVa surrounding the inserted SCC*mec* type V in S. epidermidis MB1143 shared 99% nucleotide identity with the ACME IVa in S. epidermidis MB1709. The SCC*mec* type V in MB1143 shared 97% nucleotide identity with the SCC*mec* type V in S. aureus PM1 and had a truncated J1 region. Downstream of the ACME IVa in S. epidermidis MB1143 was the *ars* operon (confirmed with primers 13F-2/13R-2; Fig. S1d).

10.1128/mBio.02911-19.1 FIG S1 Confirmation of the structures of the mobile genetic elements. The key at the top lists the primer pair used to confirm the structure, along with the isolate name. Download FIG S1, PDF file, 0.8 MB. Copyright © 2020 Arora et al. 2020 Arora et al. This content is distributed under the terms of the Creative Commons Attribution 4.0 International license.

While the *sesJ* gene was encoded in ACME IVa in S. epidermidis MB1143 and MB1709, ST5 methicillin-resistant S. epidermidis MB2193 contained the *sesJ* gene in ACME V. Unlike other isolates, in which the MGE containing the *sesJ* gene was inserted directly into the *orfX* gene, ACME V containing the *sesJ* gene was present downstream of SCC*mec* type IV (Fig. 1 and 3B) (confirmed with primers 1F/1R and 4F/14R; Fig. S1e). SCC*mec* type IV in MB2193 shared 99% nucleotide sequence identity with SCC*mec* type IVa (S. aureus CA05) and was demarcated by DR_E and DR_B. The presence of *mec* complex B and *ccr* gene complex 2 in SCC*mec* type IV in MB2193 was confirmed with primers 2F/2R and 3F/3R (Fig. S1e). DR_B also marked the left junction of the ACME V containing the *sesJ* gene, the *kdp* operon, the *arc* operon, and the *opp* operon (confirmed with primer pairs 4F/14R, 8F/8R, and 9F/9R; Fig. S1e). ACME V in S. epidermidis MB2193 shared 99% nucleotide identity with the previously reported ACME V in S. epidermidis PS19PH. Similar to ACME V in S. epidermidis PS19PH, the CRISPR element (demarcated by DR_K and DR_C) and the *ars* operon (demarcated by DR_L and DR_A) were identified downstream of ACME V in S. epidermidis MB2193 (confirmed with primer pairs 11F/11R and 13F/13R, respectively; Fig. S1e).

### Organization of the pseudo element containing the *sesJ* gene.

S. epidermidis MB1715 contained a unique MGE with the *sesJ* gene but lacked the *mecA* gene, the *ccr* genes, the *arc* operon, the *opp* operon, and the *kdp* operon (Fig. 1). Based on the guidelines by the International Working Group on the Classification of Staphylococcal Cassette Chromosome Elements, we named this element ΨSCC*sesJ* because of the presence of the *sesJ* gene. ΨSCC*sesJ* was inserted into the *orfX* gene (confirmed with primers 1F/14R; Fig. S1e) and was demarcated by DR_B and DR_A. It showed a high degree of nucleotide identity with the sequence of ACME containing the *sesJ* gene in MB1709 but lacked both the *arc* and the *kdp* operons (confirmed with primers 20F/17R; Fig. S1e). The two operons were instead replaced with five unique ORFs also found in SCC*mec* type V. Like other isolates, ΨSCC*sesJ* also contained the *ars* operon (confirmed with primers 13F-2/13R-2; Fig. S1e).

### Characterization of gene clusters containing *sesJ*.

We noticed a difference in the genes surrounding the *sesJ* gene when accompanied by DR_B or DR_E, i.e., when present in the SCC element or ACME, respectively. When accompanied by DR_B, the *sesJ* gene was present in a cluster of eight genes, referred to here as cluster 1. Cluster 1 included the *sesJ* gene, the two glycosyltransferase genes *gtfA* and *gtfB*, and five hypothetical proteins. The presence of the glycosyltransferase genes *gtfA* and *gtfB* was confirmed with primers 5F/5R (Fig. S1a and b). As mentioned earlier, cluster 1 forms the J3 region of SCC*mec* type IV in S. epidermidis MB1569 and MB1048. Similarly, cluster 1 was also present in SCC*sesJ* in S. epidermidis MB567 (Fig. 1) and shared 99% nucleotide identity with the cluster 1 in MB1048 and MB1569.

When accompanied by DR_E, the *sesJ* gene was present in a cluster of 10 genes, referred to here as cluster 2. Cluster 2 also contained the two glycosyltransferase genes *gtfA* and *gtfB* (Fig. 1) present in cluster 1 (confirmed with primers 5F/5R; Fig. S1c to e). However, the *sesJ*, *gtfA*, and *gtfB* genes in this cluster were also accompanied by the *hsdR*, *hsdS*, and *hsdM* genes, encoding a type 1 restriction modification system (confirmed with primer pairs 6F/6R and 7F/7R; Fig. S1c to e). The remaining four ORFs in cluster 2 encoded hypothetical proteins. The clusters 2 encoded by S. epidermidis isolates MB2193, MB1143, MB1715, and MB1709 shared 99% nucleotide identity with each other. When comparing the two clusters, the only shared genes were the gene upstream of *sesJ* encoding a hypothetical protein, the *sesJ* gene, and the two glycosyltransferase genes *gtfA* and *gtfB*. Interestingly, the two glycosyltransferases GtfA and GtfB share a low amino acid sequence identity with the staphylococcal glycosyltransferases encoded in the core genome (Table S2) and likely have a different origin.

### SesJ isoforms.

We have previously reported that the SesJ protein is a prototype of a novel N-terminal repeat-containing subfamily of MSCRAMMs (28). MSCRAMMs bind to their ligands through the A region, located near the N terminus (31). Different isoforms of the MSCRAMM have been identified, based on the sequence variation in the A region. For example, fibronectin binding protein A (FnbpA) has seven isoforms based on the A region itself (32). We compared the amino acid sequence of the A region of SesJ from the 7 different isolates and identified two isoforms which shared 95% identity to each other (Fig. S2 and Table S3). As expected, the SCC elements and ACME carried different isoforms of the SesJ proteins (Table 4). Furthermore, we compared the SesJ A-region sequence from 30 *sesJ*^+^ S. epidermidis isolates from this study and found that all ST2 (CC2) isolates contained isoform I and that all ST5 (CC5) and ST210 (CC5) isolates contained isoform II (Fig. S2 and Table S3). We therefore expect that all ST2 isolates contain the *sesJ* gene in the SCC element and that all ST5 and ST210 isolates contain the *sesJ* gene in ACME.

**TABLE 4:** SesJ isoforms are present in different clonal complexes

ST	CC	Isoform	MGE containing *sesJ* gene
ST2	CC2	I	SCC*mec*
ST5	CC5	II	ACME
ST210	CC5	II	ACME

10.1128/mBio.02911-19.2 FIG S2 Amino acid sequence comparison of SesJ A regions from 30 S. epidermidis isolates. Download FIG S2, PDF file, 0.03 MB. Copyright © 2020 Arora et al. 2020 Arora et al. This content is distributed under the terms of the Creative Commons Attribution 4.0 International license.

### SesJ ligand.

**FIG 4:**
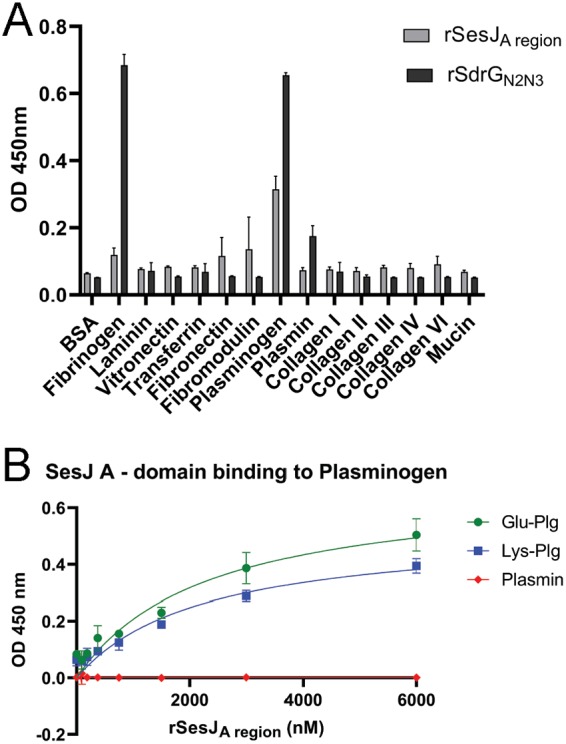
The SesJ_A region_ binds to plasminogen. (A) Ligand screen with immobilized human extracellular matrix and plasma proteins. (B) Concentration-dependent binding of rSesJ_A region_ to immobilized Glu-Plg, Lys-Plg, and plasmin. OD, optical density.

MSCRAMMs often bind host proteins present in blood and the extracellular matrix through their A region, the minimum ligand-binding region of an MSCRAMM (31). Therefore, we purified the recombinant A region of SesJ and tested its binding to a set of potential targets, including fibrinogen, fibronectin, fibromodulin, vitronectin, transferrin, mucin, plasmin, collagen I, collagen II, collagen III, collagen IV, collagen VI, plasminogen, and laminin. The recombinant SesJ A domain (rSesJ_A region_) bound to immobilized plasminogen but not to any of the other proteins tested (Fig. 4A). SdrG, another MSCRAMM of S. epidermidis, has been reported to bind human fibrinogen using N2 and N3 domains, a subregion of the A domain (33). Therefore, as expected, recombinant SdrG N2 and N3 (rSdrG_N2N3_) bound human fibrinogen. Next, we examined the concentration dependence of rSesJ_A region_ (at concentrations ranging from 93 nM to 6 μM) binding to Glu-plasminogen (Glu-Plg), the native form of the zymogen, and Lys-plasminogen (Lys-Plg), the intermediate plasminogen in the conversion of Glu-Plg to plasmin. rSesJ_A region_ bound Glu-Plg and Lys-Plg with apparent equilibrium dissociation constant values of 2.2 μM and 2.3 μM, respectively, but not plasmin (Fig. 4B).

## DISCUSSION

S. epidermidis is increasingly being recognized as a serious, opportunistic pathogen. However, its pathogenic mechanisms beyond biofilm formation are largely unknown. Furthermore, most of the identified virulence factors are encoded in the core genome, whereas 20% of the S. epidermidis genome consists of variable genes that may alter the pathogenic potential of the isolate (34). In an attempt to expand our knowledge of S. epidermidis pathogenesis, we aimed to identify novel cell wall-anchored proteins, which led to the discovery of the SesJ protein.

In our study of the S. epidermidis clinical isolates, we found that the *sesJ* gene was present in 18% of the isolates. Although the current study was limited to one hospital in the United States, we previously reported on an epidemiology study for the *sesJ* gene with isolates collected at Columbia University Medical Center (28). In the previous study, we investigated isolates from patients with an infected left ventricular assist driveline (28). We observed similar percentages of *sesJ*^+^ isolates in both studies.

In addition to the prevalence, we aimed to characterize the presence of the *sesJ* gene in different STs. Multiple STs were observed among the 171 S. epidermidis isolates. ST5, ST2, and ST83 isolates accounted for over 60% of the total isolates tested. ST2 is one of the most successful genetic lineages of pathogenic S. epidermidis spread worldwide (4, 6, 7, 35–37). Recent reports have documented a higher proportion of ST5 than ST2 among S. epidermidis isolates causing bloodstream infections and bone and joint infections (7, 37, 38). Similarly, ST5 clinical isolates predominated in this study. ST83 is not as widespread and predominant as ST2 and ST5, but ST83 has been observed in the United States, Italy, and Greece (7, 18, 39). We also observed ST210 among the bloodstream isolates. In a study from China, ST210 was associated with health care personnel only and was absent among the isolates obtained from patients (35). However, we are starting to observe more reports of ST210 isolates causing infections in patients. Interestingly, all ST210 isolates in our study encoded the *sesJ* gene. Additionally, three ST210 S. epidermidis isolates have been sequenced and deposited in a public database as a part of two different studies in the past 2 years (24, 40). Two of these ST210 isolates were collected from the bloodstreams of patients in Australia with at least two positive blood cultures (40). A third ST210 isolate was collected from an intraoral site of a patient with periodontal diseases in Ireland (24). We analyzed the genomes of these ST210 isolates and identified these to be *sesJ*^+^ as well. Possibly, S. epidermidis ST210 isolates can cause infection upon acquiring potential virulence factors like SesJ.

The *sesJ* gene is part of the accessory genome and encoded in an MGE. In fact, SesJ is the first MSCRAMM encoded on an MGE. In our study, we found that the *sesJ* gene can be present in an SCC element as well as in an ACME. Coagulase-negative staphylococci act as a reservoir of virulence factors that can be transferred through horizontal gene transfer to other staphylococcal species, like S. aureus (41). The presence of the *sesJ* gene in an MGE raises the possibility of its transfer to S. aureus. Another CWA protein, called Pls, has been reported in the J region of SCC*mec* type 1 and ΨSCC*pls* in S. aureus and in an SCC element in S. epidermidis (25, 42, 43). In our study, we observed the presence of the *sesJ* gene in ACME IV and ACME V. Previous work by O’Connor et al. on the prevalence of ACME in S. epidermidis also reported the presence of different CWA proteins in MGEs. In their studies, the SesJ protein was properly sequenced, but it was misannotated as SdrH (24).

In this collection, we observed two isoforms of SesJ with 95% identity in the A region. Isoform I is encoded in an SCC element present in ST2 and ST218 isolates. Isoform II is encoded in ACME present in ST5 and ST210 isolates. The A region is the ligand-binding region of the MSCRAMM that evolves through point mutations. The most notable difference in the isoform function has been observed for the MSCRAMM called Bbp, which is an isoform of SdrE. The A regions of Bbp and SdrE share about 70% amino acid identity, but the two proteins have a 5-fold difference in their binding affinities to human fibrinogen (44). Seven different isoforms of SdrD have been reported based on the sequence differences in the A region of SdrD in S. aureus isolates from healthy nasal carriers (45). MSCRAMM FnbpA binds human fibrinogen and fibronectin through the A region and fibronectin binding repeats, respectively. A recent study showed that some S. aureus isolates causing cardiovascular device infections in patients encode FnbpA with amino acid polymorphisms in fibronectin binding repeats. These amino acid polymorphisms significantly affect the bond strength between FnbpA and fibronectin and are correlated with distinct infections caused by S. aureus (46–48). Additionally, based on the amino acid sequence of the A region, multiple isoforms of FnbpA and FnbpB have been identified (32, 49).

Two glycosyltransferase genes were observed adjacent to the *sesJ* gene. Glycosyltransferase genes in close proximity to Pls genes and genes for other MSCRAMMs have been observed before (26). Two glycosyltransferase genes, *sdgA* and *sdgB*, are encoded by a region adjacent to the *sdrC*, *sdrD*, and *sdrE* gene cluster in S. aureus. Both SdgA and SdgB covalently link *N*-acetylglucosamine (GlcNAc) to the serine-aspartate dipeptide (SD) repeats of MSCRAMM proteins SdrC, SdrD, SdrE, ClfA, and ClfB. GlcNAc modification of these MSCRAMM proteins acts as dual-edge sword. On the one hand, it resulted in protection of these proteins from proteolysis by human neutrophil-derived cathepsin G and possibly other proteases. On the other hand, glycosylation created an immunodominant epitope for a strong antibody response (50). Similarly, genes for the two glycosyltransferases GtfC and GtfD were identified downstream of the *pls* gene in the SCC*mec* element. GtfC and GtfD add *N*-acetylhexosamine to the serine residues in SD repeats of the Pls protein. Sugar modification of Pls by GtfC and GtfD leads to enhanced biofilm formation (26). We hypothesize that the SesJ protein on the surface of S. epidermidis is glycosylated by GtfA and GtfB. Studies to determine the glycosylation of SesJ are in progress.

Lastly, we explored the ability of SesJ to bind to different blood and matrix proteins. Gram-positive pathogens causing bloodstream infections often survive in the host blood by manipulating the coagulation or fibrinolytic pathways (33, 51–53). Since SesJ is present in an MGE in S. epidermidis bloodstream isolates, we speculated that SesJ might target a blood protein. In fact, SesJ does bind plasminogen in a concentration-dependent manner. Plasminogen is a 92-kDa zymogen precursor of plasmin, a serine protease that degrades fibrin (54). The degradation of fibrin is a key step in fibrinolysis (54). Bacterial proteins recruit plasminogen onto the surface of the bacteria, where it is activated to plasmin by tissue plasminogen activator and urokinase plasminogen activator (55, 56). In addition, virulence factors like staphylokinase and streptokinase activate plasminogen to plasmin (51, 53). Pathogens then utilize activated plasmin for both invasion and immune evasion (55, 56). However, SesJ binds both Glu-Plg and Lys-Plg with a modest affinity, and SesJ does not appear to bind to plasmin. Plasminogen binds to lysine residues in a number of proteins in a rather nonspecific interaction. The cell wall-anchored proteins represent the major molecular interface between a Gram-positive bacterium and its environment. MSCRAMMs constitute a family of structurally related CWA proteins that often bind their ligand though variants of a dynamic mechanism that we have called dock, lock, and latch (DLL) (31, 57, 58). Sequence analysis and structural modeling suggest that SesJ contains all the features involved in the DLL binding process (28). Consequently, we speculate that this binding mechanism is also in play for SesJ. A key step in the DLL mechanism is the docking of a linear segment of the targeted ligand to a trench formed between the two subdomains. This interaction seems to be rather weak, and ongoing studies in the Hook lab show that this weak docking interaction can be complemented by secondary interactions, which, combined with the initial docking, result in an overall high-affinity interaction. Whether SesJ binding to plasminogen follows this model is unclear at present, and substantial additional work is needed to determine the significance of the SesJ-plasminogen interaction. Alternative roles for SesJ in the pathogenic process of invasive S. epidermidis strains are also being considered in ongoing studies.

## MATERIALS AND METHODS

### Isolates and antibiotic resistance typing.

The S. epidermidis clinical isolates used in this study and their antibiotic resistance typing have been described before by Li et al. (29). In short, isolates were collected from the blood of patients that presented with symptoms of bloodstream infections at the MD Anderson Cancer Center, Houston, TX. The isolates were collected from 2013 to 2016 with the approval of the MD Anderson Cancer Center Institutional Review Board (approval number PA16-0066) and stored for future analysis. Matrix-assisted laser desorption ionization–time of flight mass spectrometry was used to confirm that the isolates belonged to S. epidermidis species.

### MLST and clonal complex determination.

Multilocus sequence typing (MLST) and clonal complex determination were done by Li et al. (29). In summary, the draft genome assemblies from Illumina reads were used to determine the multilocus sequence type by batch sequence query of the Bacterial Isolate Genome Sequence Database (BIGSdb) (59). Clonal complexes were determined by using the eBURST algorithm (60).

### Library preparation and genome sequencing.

Minion sequencing and library preparation were done at the Institute for Genome Sciences and Society, Texas A&M University. Bacterial isolates were grown overnight in 5 ml LB medium at 37°C and 200 rpm. Overnight cultures were centrifuged at 4,000 rpm for 10 min. Bacterial pellets were washed twice with phosphate-buffered saline (PBS) buffer. For Minion sequencing, genomic DNA (gDNA) was extracted from the bacterial pellets using a Macherey Nagle NucleoMag tissue kit and NucleoMag B beads. Sample lysis was performed using both proteinase K and RNase A, as suggested by the manufacturer. Next, a Qubit double-stranded DNA BR assay kit and Qubit fluorometer were used to measure DNA quantity. Four hundred nanograms of the gDNA was used for barcoding and library preparation using a rapid barcoding kit from Nanopore Technologies, with the exception that adaptors were added to the tagged ends. An Oxford Nanopore DNA sequencer was used for long-read sequencing. Illumina and Pacific Biosciences (PacBio) sequencing for these isolates has been described by Li et al. (29). To summarize, genomic DNA for Illumina sequencing was extracted using a MasterPure kit (Illumina, Inc., San Diego, CA). Ten micrograms of gDNA was used for paired-end sequencing on the Illumina instrument (Illumina, Inc., San Diego, CA), using TruSeq chemistry, at the MD Anderson Sequencing and Microarray Facility. gDNA for PacBio sequencing was extracted using phenol-chloroform extraction from overnight cultures. The Pacific Biosciences guidelines for preparing a 20-kb SMRTbell template were used to create a large insert library.

### Genome assembly.

The quality of the reads generated through Illumina and Minion sequencing was assessed using the FastQC tool kit (Babraham Bioinformatics). Adaptors as well as low-quality Illumina reads were trimmed using the Trimmomatic (v0.33) tool, as reported by Li et al. (29). Nanopore reads were demultiplexed with the Albacore pipeline. Hybrid assembly, using Illumina and Nanopore reads, was generated using the normal mode for the Unicycler pipeline for S. epidermidis isolates MB1048, MB1569, MB1715, MB1709, and MB567 (61). For the S. epidermidis MB2193 and MB1143 isolates, the hierarchical genome assembly process pipeline was implemented for the *de novo* genome assembly of PacBio reads (62). Next, PacBio assembly results were corrected by mapping paired-end Illumina short reads using the Bowtie2 (v2.2.3) program (63). The CLC Main Workbench was used to generate FASTA files for the MGEs.

### MGE identification.

MGEs were identified *in silico* using the characteristic features specific to the MGE. The SCC*mec* type was determined *in silico* by identifying the combination of the *mec* complex class and the type of *ccr* complex. Different *mec* gene complexes and *ccr* gene combinations were queried in the assembled genome sequence data for all the study isolates. The presence of the *mec* gene complex and the *ccr* gene complex was further confirmed by visualization of the read alignment for the genome. The structure of the MGEs was also confirmed by PCR using the primers listed in Table S1 in the supplemental material. The Emerald GT PCR master mix or Phusion *Taq* polymerase was used for PCRs, following the manufacturer’s recommended protocol. DRs were identified using the inbuilt Find Repeat and Emboss tool fuzznuc in Geneious software. Easyfig (v2.2.2) software was used to generate the figures showing structural comparisons and the nucleotide identities of the different MGEs (64).

10.1128/mBio.02911-19.3 TABLE S1 Primers used for confirming MGE structures. Download Table S1, PDF file, 0.1 MB. Copyright © 2020 Arora et al. 2020 Arora et al. This content is distributed under the terms of the Creative Commons Attribution 4.0 International license.

10.1128/mBio.02911-19.4 TABLE S2 Amino acid identity comparison of SesJ-associated GtfA and GtfB with other staphylococcal glycosyltransferases encoded in the core genome. Download Table S2, PDF file, 0.05 MB. Copyright © 2020 Arora et al. 2020 Arora et al. This content is distributed under the terms of the Creative Commons Attribution 4.0 International license.

10.1128/mBio.02911-19.5 TABLE S3 Amino acid identities of the SesJ A region from 30 S. epidermidis isolates. Download Table S3, XLSX file, 0.01 MB. Copyright © 2020 Arora et al. 2020 Arora et al. This content is distributed under the terms of the Creative Commons Attribution 4.0 International license.

### Identification of *sesJ* and *gtf* genes.

The sequence of the *sesJ* gene was deposited in a public database as part of the previous study (28). For the *gtfA* and *gtfB* genes, the nucleotide sequences were obtained from the finished genomes and used for further analysis. The nucleotide sequences of the *sesJ*, *gtfA*, and *gtfB* genes were used to query the local database containing the assembled genome sequence data for the study isolates. The presence of the genes was confirmed by visualization of the read alignment for the genome. For the 7 isolates for which the full genome was assembled and MGEs were identified, the genes were also confirmed by PCR using the primers listed in Table S1.

### Recombinant SesJ protein.

The recombinant SesJ A domain (rSesJ_A region_) was generated and purified as described earlier (28).

### ELISA-based binding assays.

An Immulon 4HBX 96-well plate was coated with 500 ng/well of ligands in PBS, pH 7.5, for 1 h at room temperature (RT). The plate was washed three times with PBS, 0.05% Tween 20 (PBST) and blocked with 3% (wt/vol) bovine serum albumin (BSA) for 1 h at RT. The plate was washed three times with PBST; 5 μM rSesJ_A region_, diluted into 1% BSA and PBST, was added to the wells; and the plate was incubated for 1 h at room temperature. The plate was washed three times with PBST, and the bound rSesJ_A region_ was detected by adding a 1:3,000 dilution of an anti-His tag horseradish peroxidase-conjugated antibody in PBST, 1% BSA. The plate was incubated for 1 h at room temperature and then washed three times with PBST. The plate was developed with *o*-phenylenediamine dihydrochloride tablets (Sigma Fast) at room temperature for ∼30 min. The plates were read at 450 nm using an enzyme-linked immunosorbent assay (ELISA) plate reader (ThermoMax microplate reader; Molecular Devices). The same method outlined above was followed for the plasminogen binding assays, except that the microtiter wells were coated with 1 μg/well of ligand proteins and the wells were incubated with different concentrations of the rSesJ_A region_.

### Data availability.

The MGEs from S. epidermidis MB567 (GenBank accession number MK770829), MB1048 (GenBank accession number MK778453), MB2193 (GenBank accession number MK784553), MB1715 (GenBank accession number MK784554), MB1143 (GenBank accession number MK784555), MB1709 (GenBank accession number MK784556) and MB1569 (GenBank accession number MK784557) described here have been deposited in GenBank.
