# Supplementary material for: Staphylococcus epidermidis MSCRAMM SesJ Is Encoded in Composite Islands
Source: mBio. 2020 Feb 18;11(1):e02911-19. doi: 10.1128/mBio.02911-19 (PMC7029136; doi:10.1128/mBio.02911-19)
Supplement: FIG S1 [file mBio.02911-19-sf001.pdf]

Figure S1: Confirmation of the structures of mobile genetic elements. Legend on the top lists the primer pair used to confirm the structure, along with the isolate name.

(a)

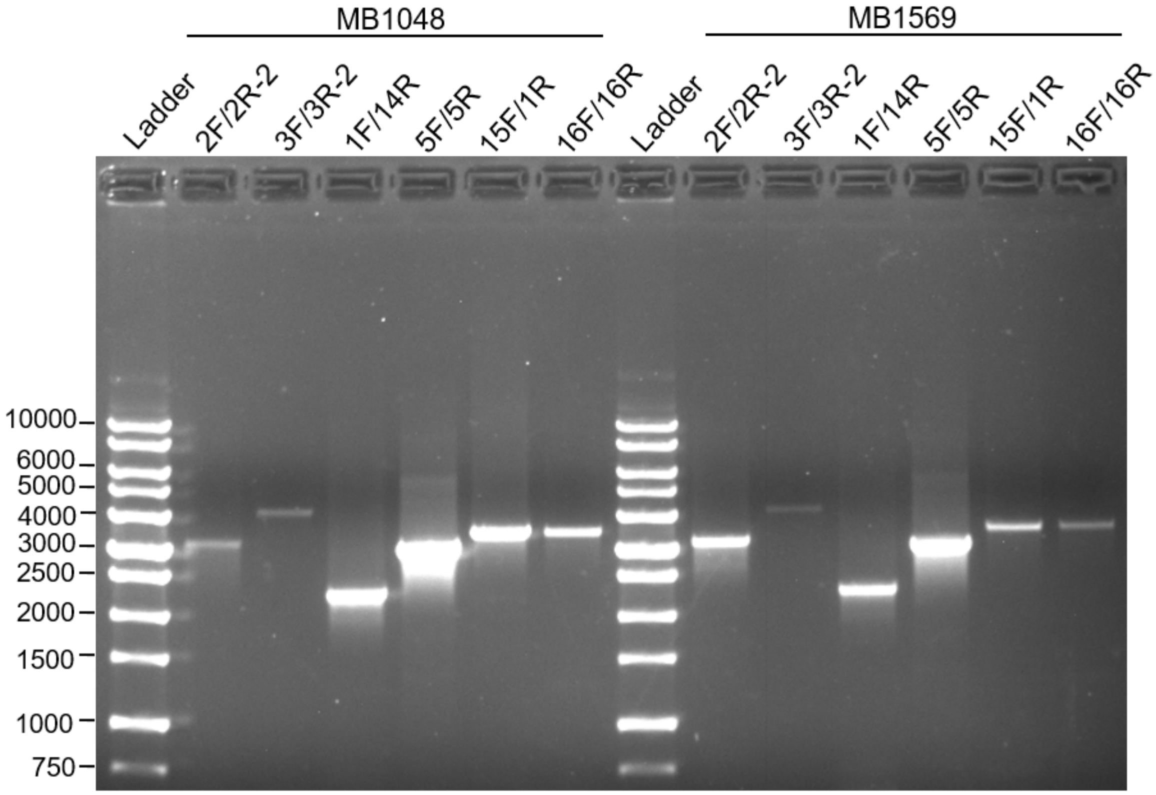

(b)

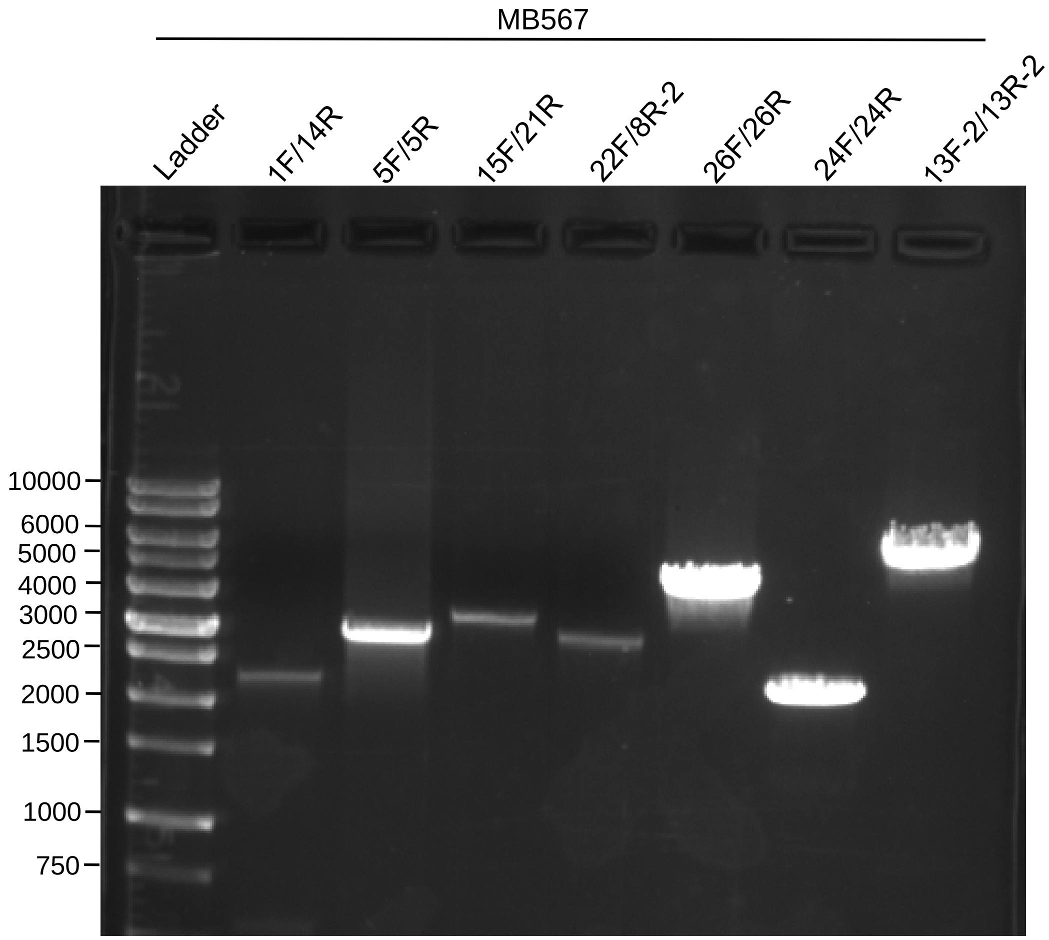
MB567
Ladder
1F/14R
5F/5R
15F/21R
22F/8R-2
26F/26R
24F/24R
13F-2/13R-2
10000
6000
5000
4000
3000
2500
2000
1500
1000
750

(c)

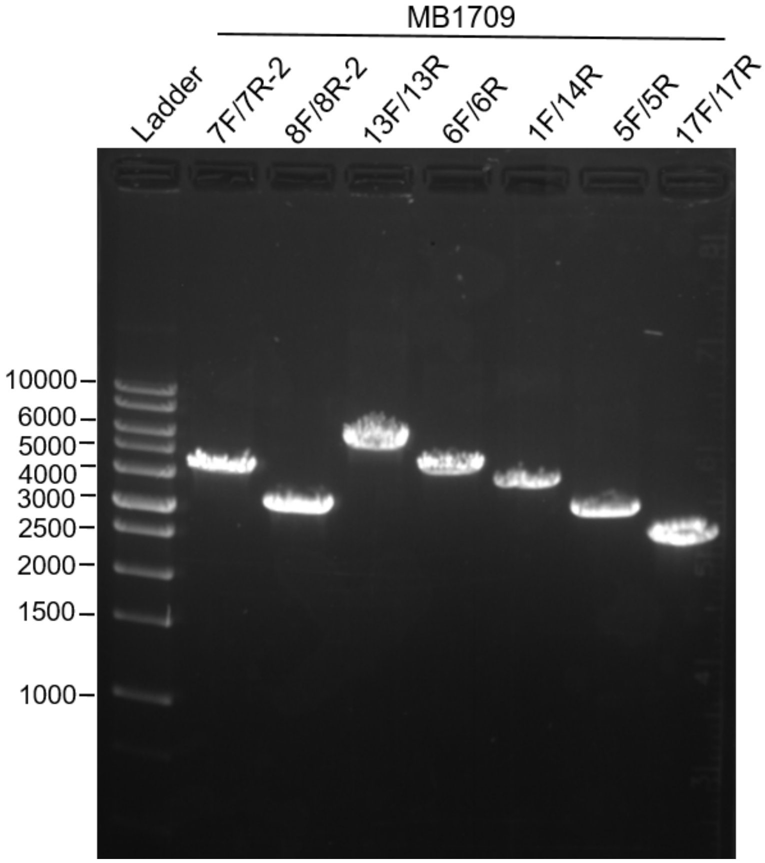

MB1709
Ladder
7F/7R-2
8F/8R-2
13F/13R
6F/6R
1F/14R
5F/5R
17F/17R
10000
6000
5000
4000
3000
2500
2000
1500
1000

(d)

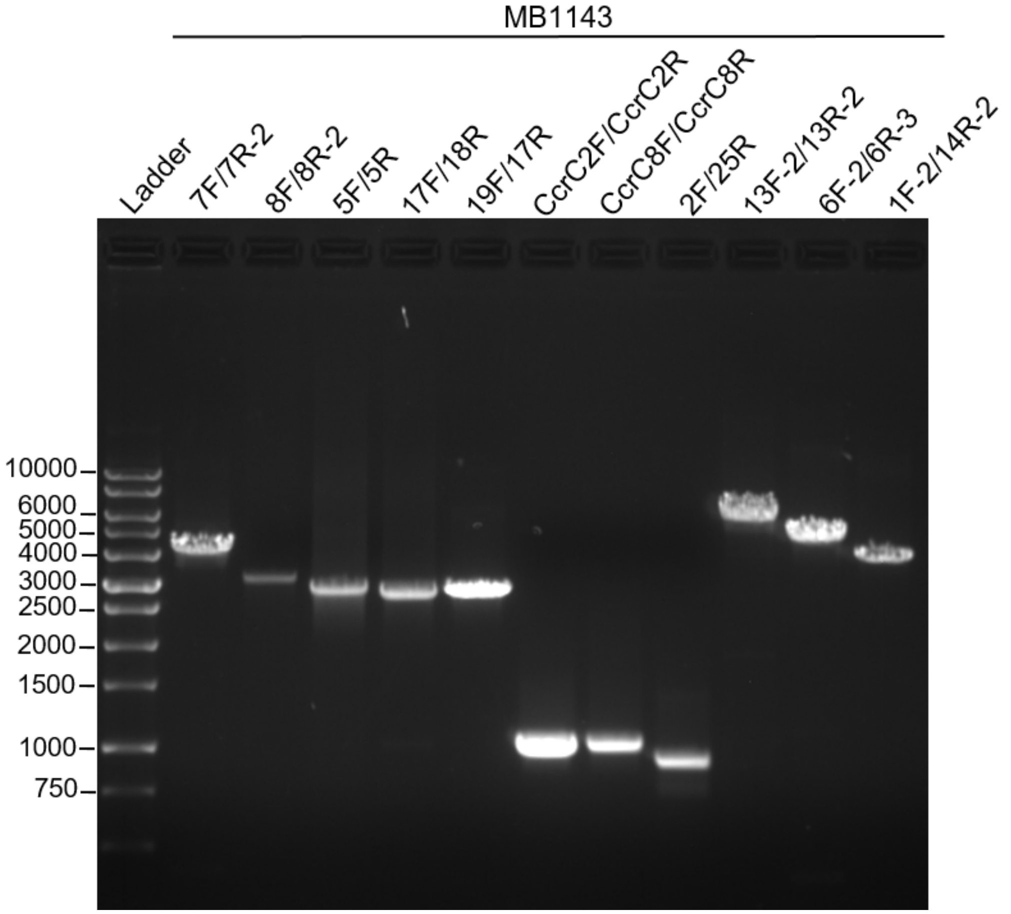
MB1143
Ladder
7F/7R-2
8F/8R-2
5F/5R
17F/18R
19F/17R
CcrC2F/CcrC2R
CcrC8F/CcrC8R
2F/25R
13F-2/13R-2
6F-2/6R-3
1F-2/14R-2
10000
6000
5000
4000
3000
2500
2000
1500
1000
750

(e)

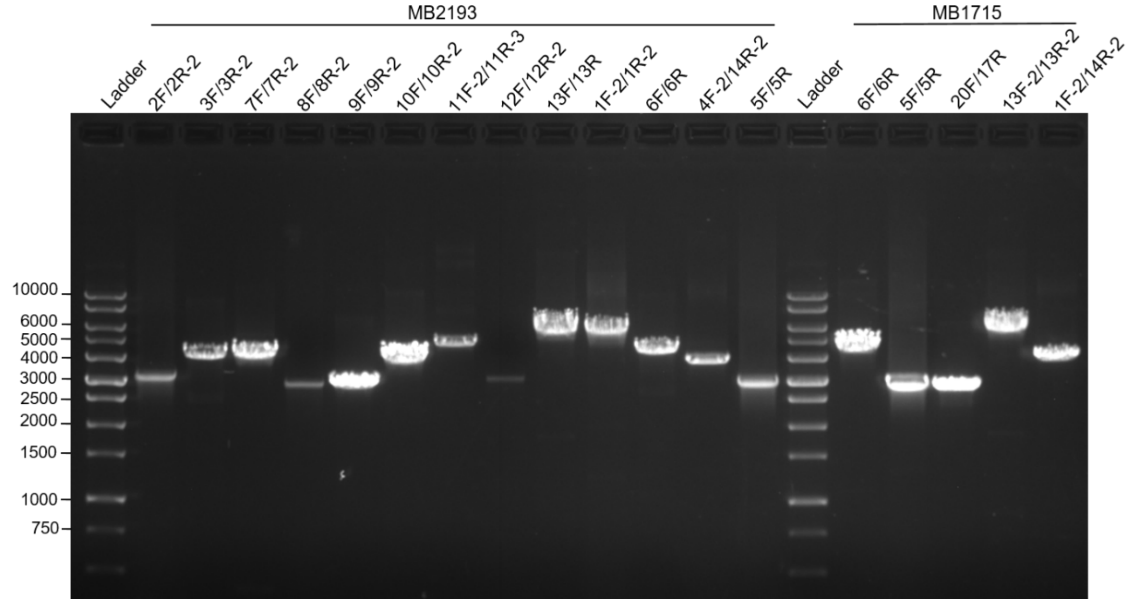
MB2193
MB1715
Ladder
2F/2R-2
3F/3R-2
7F/7R-2
8F/8R-2
9F/9R-2
10F/10R-2
11F-2/11R-3
12F/12R-2
13F/13R
1F-2/1R-2
6F/6R
4F-2/14R-2
5F/5R
Ladder
6F/6R
5F/5R
20F/17R
13F-2/13R-2
1F-2/14R-2
10000
6000
5000
4000
3000
2500
2000
1500
1000
750
