# Supplementary material for: Staphylococcus epidermidis MSCRAMM SesJ Is Encoded in Composite Islands
Source: mBio. 2020 Feb 18;11(1):e02911-19. doi: 10.1128/mBio.02911-19 (PMC7029136; doi:10.1128/mBio.02911-19)
Supplement: FIG S2 [file mBio.02911-19-sf002.pdf]

Figure S2: Amino acid sequence comparison of SesJ A-region from 30 *S. epidermidis* isolates.

```
MB905_ST5          PEVDSEVLDNSKQGTDNDNSSNHSEDNIVTYAEPTALTNTRSVDSPSRVSSDNSQAQKQG   60
MB1143_ST210       PEVDSEVLDNSKQGTDNDNSSNHSEDNIVTYAEPTALTNTRSVDSPSRVSSDNSQAQKQG   60
MB1312_St5         PEVDSEVLDNSKQGTDNDNSSNHSEDNIVTYAEPTALTNTRSVDSPSRVSSDNSQAQKQG   60
MB1709_ST210       PEVDSEVLDNSKQGTDNDNSSNHSEDNIVTYAEPTALTNTRSVDSPSRVSSDNSQAQKQG   60
MB1715_ST210       PEVDSEVLDNSKQGTDNDNSSNHSEDNIVTYAEPTALTNTRSVDSPSRVSSDNSQAQKQG   60
MB1907_ST210       PEVDSEVLDNSKQGTDNDNSSNHSEDNIVTYAEPTALTNTRSVDSPSRVSSDNSQAQKQG   60
MB526_ST210        PEVDSEVLDNSKQGTDNDNSSNHSEDNIVTYAEPTALTNTRSVDSPSRVSSDNSQAQKQG   60
MB896_ST5          PEVDSEVLDNSKQGTDNDNSSNHSEDNIVTYAEPTALTNTRSVDSPSRVSSDNSQAQKQG   60
MB2193_ST5         PEVDSEVLDNSKQGTDNDNSSNHSEDNIVTYAEPTALTNTRSVDSPSRVSSDNSQAQKQG   60
MB740_ST5          PEVDSEVLDNSKQGTDNDNSSNHSEDNIVTYAEPTALTNTRSVDSPSRVSSDNSQAQKQG   60
MB972_ST5          PEVDSEVLDNSKQGTDNDNSSNHSEDNIVTYAEPTALTNTRSVDSPSRVSSDNSQAQKQG   60
MB1037_ST5         PEVDSEVLDNSKQGTDNDNSSNHSEDNIVTYAEPTALTNTRSVDSPSRVSSDNSQAQKQG   60
MB1064_ST5         PEVDSEVLDNSKQGTDNDNSSNHSEDNIVTYAEPTALTNTRSVDSPSRVSSDNSQAQKQG   60
MB1489_ST5         PEVDSEVLDNSKQGTDNDNSSNHSEDNIVTYAEPTALTNTRSVDSPSRVSSDNSQAQKQG   60
MB1586_ST210       PEVDSEVLDNSKQGTDNDNSSNHSEDNIVTYAEPTALTNTRSVDSPSRVSSDNSQAQKQG   60
MB1679_ST5         PEVDSEVLDNSKQGTDNDNSSNHSEDNIVTYAEPTALTNTRSVDSPSRVSSDNSQAQKQG   60
MB1691a_ST5        PEVDSEVLDNSKQGTDNDNSSNHSEDNIVTYAEPTALTNTRSVDSPSRVSSDNSQAQKQG   60
MB988_ST5          PEVDSEVLDNSKQGTDNDNSSNHSEDNIVTYAEPTALTNTRSVDSPSRVSSDNSQAQKQG   60
MB1695_ST5         PEVDSEVLDNSKQGTDNDNSSNHSEDNIVTYAEPTALTNTRSVDSPSRVSSDNSQAQKQG   60
MB1961_ST5         PEVDSEVLDNSKQGTDNDNSSNHSEDNIVTYAEPTALTNTRSVDSPSRVSSDNSQAQKQG   60
MB383_ST5          PEVDSEVLDNSKQGTDNDNSSNHSEDNIVTYAEPTALTNTRSVDSPSRVSSDNSQAQKQG   60
MB10_ST5           PEVDSEVLDNSKQGTDNDNSSNHSEDNIVTYAEPTALTNTRSVDSPSRVSSDNSQAQKQG   60
MB1048_ST2         PEVDSEVLDNSKQGTDNDNSSNHSEDNIVTYAEPTALTNTRSVDSPSRVSSDNSQAQKQG   60
MB1299_ST57        PEVDSEVLDNSKQGTDNDNSSNHSEDNIVTYAEPTALTNTRSVDSPSRVSSDNSQAQKQG   60
MB1306_ST2         PEVDSEVLDNSKQGTDNDNSSNHSEDNIVTYAEPTALTNTRSVDSPSRVSSDNSQAQKQG   60
MB1651_ST2         PEVDSEVLDNSKQGTDNDNSSNHSEDNIVTYAEPTALTNTRSVDSPSRVSSDNSQAQKQG   60
MB1750_ST2         PEVDSEVLDNSKQGTDNDNSSNHSEDNIVTYAEPTALTNTRSVDSPSRVSSDNSQAQKQG   60
MB567_ST218        PEVDSEVLDNSKQGTDNDNSSNHSEDNIVTYAEPTALTNTRSVDSPSRVSSDNSQAQKQG   60
MB1569_ST2         PEVDSEVLDNSKQGTDNDNSSNHSEDNIVTYAEPTALTNTRSVDSPSRVSSDNSQAQKQG   60
MB1985_ST2         PEVDSEVLDNSKQGTDNDNSSNHSEDNIVTYAEPTALTNTRSVDSPSRVSSDNSQAQKQG   60
                   ************************************************************

MB905_ST5          KNVNDSIKVNSVDTDKEYVEPNNGQGFSTNVSFEVDGKVNKGDYFTVDMPEYADFNGIAD   120
MB1143_ST210       KNVNDSIKVNSVDTDKEYVEPNNGQGFSTNVSFEVDGKVNKGDYFTVDMPEYADFNGIAD   120
MB1312_St5         KNVNDSIKVNSVDTDKEYVEPNNGQGFSTNVSFEVDGKVNKGDYFTVDMPEYADFNGIAD   120
MB1709_ST210       KNVNDSIKVNSVDTDKEYVEPNNGQGFSTNVSFEVDGKVNKGDYFTVDMPEYADFNGIAD   120
MB1715_ST210       KNVNDSIKVNSVDTDKEYVEPNNGQGFSTNVSFEVDGKVNKGDYFTVDMPEYADFNGIAD   120
MB1907_ST210       KNVNDSIKVNSVDTDKEYVEPNNGQGFSTNVSFEVDGKVNKGDYFTVDMPEYADFNGIAD   120
MB526_ST210        KNVNDSIKVNSVDTDKEYVEPNNGQGFSTNVSFEVDGKVNKGDYFTVDMPEYADFNGIAD   120
MB896_ST5          KNVNDSIKVNSVDTDKEYVEPNNGQGFSTNVSFEVDGKVNKGDYFTVDMPEYADFNGIAD   120
MB2193_ST5         KNVNDSIKVNSVDTDKEYVEPNNGQGFSTNVSFEVDGKVNKGDYFTVDMPEYADFNGIAD   120
MB740_ST5          KNVNDSIKVNSVDTDKEYVEPNNGQGFSTNVSFEVDGKVNKGDYFTVDMPEYADFNGIAD   120
MB972_ST5          KNVNDSIKVNSVDTDKEYVEPNNGQGFSTNVSFEVDGKVNKGDYFTVDMPEYADFNGIAD   120
MB1037_ST5         KNVNDSIKVNSVDTDKEYVEPNNGQGFSTNVSFEVDGKVNKGDYFTVDMPEYADFNGIAD   120
MB1064_ST5         KNVNDSIKVNSVDTDKEYVEPNNGQGFSTNVSFEVDGKVNKGDYFTVDMPEYADFNGIAD   120
MB1489_ST5         KNVNDSIKVNSVDTDKEYVEPNNGQGFSTNVSFEVDGKVNKGDYFTVDMPEYADFNGIAD   120
MB1586_ST210       KNVNDSIKVNSVDTDKEYVEPNNGQGFSTNVSFEVDGKVNKGDYFTVDMPEYADFNGIAD   120
MB1679_ST5         KNVNDSIKVNSVDTDKEYVEPNNGQGFSTNVSFEVDGKVNKGDYFTVDMPEYADFNGIAD   120
MB1691a_ST5        KNVNDSIKVNSVDTDKEYVEPNNGQGFSTNVSFEVDGKVNKGDYFTVDMPEYADFNGIAD   120
MB988_ST5          KNVNDSIKVNSVDTDKEYVEPNNGQGFSTNVSFEVDGKVNKGDYFTVDMPEYADFNGIAD   120
MB1695_ST5         KNVNDSIKVNSVDTDKEYVEPNNGQGFSTNVSFEVDGKVNKGDYFTVDMPEYADFNGIAD   120
MB1961_ST5         KNVNDSIKVNSVDTDKEYVEPNNGQGFSTNVSFEVDGKVNKGDYFTVDMPEYADFNGIAD   120
MB383_ST5          KNVNDSIKVNSVDTDKEYVEPNNGQGFSTNVSFEVDGKVNKGDYFTVDMPEYADFNGIAD   120
MB10_ST5           KNVNDSIKVNSVDTDKEYVEPNNGQGFSTNVSFEVDGKVNKGDYFTVDMPEYADFNGIAD   120
MB1048_ST2         KNVNDSIKVNSVDTDKEYVEPNNGQGFSTNVSFEVDGKVNKGDYFTVDMPEYADFNGIAD   120
MB1299_ST57        KNVNDSIKVNSVDTDKEYVEPNNGQGFSTNVSFEVDGKVNKGDYFTVDMPEYADFNGIAD   120
MB1306_ST2         KNVNDSIKVNSVDTDKEYVEPNNGQGFSTNVSFEVDGKVNKGDYFTVDMPEYADFNGIAD   120
MB1651_ST2         KNVNDSIKVNSVDTDKEYVEPNNGQGFSTNVSFEVDGKVNKGDYFTVDMPEYADFNGIAD   120
MB1750_ST2         KNVNDSIKVNSVDTDKEYVEPNNGQGFSTNVSFEVDGKVNKGDYFTVDMPEYADFNGIAD   120
MB567_ST218        KNVNDSIKVNSVDTDKEYVEPNNGQGFSTNVSFEVDGKVNKGDYFTVDMPEYADFNGIAD   120
MB1569_ST2         KNVNDSIKVNSVDTDKEYVEPNNGQGFSTNVSFEVDGKVNKGDYFTVDMPEYADFNGIAD   120
MB1985_ST2         KNVNDSIKVNSVDTDKEYVEPNNGQGFSTNVSFEVDGKVNKGDYFTVDMPEYADFNGIAD   120
                   ************************************************************
```

```
MB905_ST5          YKAANNKIYPTINDGEQVVANGVYDTETKKLVYTFTDYVNKKDNIKGQFEIPQFIDRKNA   180
MB1143_ST210       YKAANNKIYPTINDGEQVVANGVYDTETKKLVYTFTDYVNKKDNIKGQFEIPQFIDRKNA   180
MB1312_St5         YKAANNKIYPTINDGEQVVANGVYDTETKKLVYTFTDYVNKKDNIKGQFEIPQFIDRKNA   180
MB1709_ST210       YKAANNKIYPTINDGEQVVANGVYDTETKKLVYTFTDYVNKKDNIKGQFEIPQFIDRKNA   180
MB1715_ST210       YKAANNKIYPTINDGEQVVANGVYDTETKKLVYTFTDYVNKKDNIKGQFEIPQFIDRKNA   180
MB1907_ST210       YKAANNKIYPTINDGEQVVANGVYDTETKKLVYTFTDYVNKKDNIKGQFEIPQFIDRKNA   180
MB526_ST210        YKAANNKIYPTINDGEQVVANGVYDTETKKLVYTFTDYVNKKDNIKGQFEIPQFIDRKNA   180
MB896_ST5          YKAANNKIYPTINDGEQVVANGVYDTETKKLVYTFTDYVNKKDNIKGQFEIPQFIDRKNA   180
MB2193_ST5         YKAANNKIYPTINDGEQVVANGVYDTETKKLVYTFTDYVNKKDNIKGQFEIPQFIDRKNA   180
MB740_ST5          YKAANNKIYPTINDGEQVVANGVYDTETKKLVYTFTDYVNKKDNIKGQFEIPQFIDRKNA   180
MB972_ST5          YKAANNKIYPTINDGEQVVANGVYDTETKKLVYTFTDYVNKKDNIKGQFEIPQFIDRKNA   180
MB1037_ST5         YKAANNKIYPTINDGEQVVANGVYDTETKKLVYTFTDYVNKKDNIKGQFEIPQFIDRKNA   180
MB1064_ST5         YKAANNKIYPTINDGEQVVANGVYDTETKKLVYTFTDYVNKKDNIKGQFEIPQFIDRKNA   180
MB1489_ST5         YKAANNKIYPTINDGEQVVANGVYDTETKKLVYTFTDYVNKKDNIKGQFEIPQFIDRKNA   180
MB1586_ST210       YKAANNKIYPTINDGEQVVANGVYDTETKKLVYTFTDYVNKKDNIKGQFEIPQFIDRKNA   180
MB1679_ST5         YKAANNKIYPTINDGEQVVANGVYDTETKKLVYTFTDYVNKKDNIKGQFEIPQFIDRKNA   180
MB1691a_ST5        YKAANNKIYPTINDGEQVVANGVYDTETKKLVYTFTDYVNKKDNIKGQFEIPQFIDRKNA   180
MB988_ST5          YKAANNKIYPTINDGEQVVANGVYDTETKKLVYTFTDYVNKKDNIKGQFEIPQFIDRKNA   180
MB1695_ST5         YKAANNKIYPTINDGEQVVANGVYDTETKKLVYTFTDYVNKKDNIKGQFEIPQFIDRKNA   180
MB1961_ST5         YKAANNKIYPTINDGEQVVANGVYDTETKKLVYTFTDYVNKKDNIKGQFEIPQFIDRKNA   180
MB383_ST5          YKAANNKIYPTINDGEQVVANGVYDTETKKLVYTFTDYVNKKDNIKGQFEIPQFIDRKNA   180
MB10_ST5           YKAANNKIYPTINDGEQVVANGVYDTETKKLVYTFTDYVNKKDNIKGQFEIPQFIDRKNA   180
MB1048_ST2         YKAANNKIYPTINDGEQVVANGVYDTETKKLVYTFTDYVNKKDNIKGQFEIPQFIDRKNA   180
MB1299_ST57        YKAANNKIYPTINDGEQVVANGVYDTETKKLVYTFTDYVNKKDNIKGQFEIPQFIDRKNA   180
MB1306_ST2         YKAANNKIYPTINDGEQVVANGVYDTETKKLVYTFTDYVNKKDNIKGQFEIPQFIDRKNA   180
MB1651_ST2         YKAANNKIYPTINDGEQVVANGVYDTETKKLVYTFTDYVNKKDNIKGQFEIPQFIDRKNA   180
MB1750_ST2         YKAANNKIYPTINDGEQVVANGVYDTETKKLVYTFTDYVNKKDNIKGQFEIPQFIDRKNA   180
MB567_ST218        YKAANNKIYPTINDGEQVVANGVYDTETKKLVYTFTDYVNKKDNIKGQFEIPQFIDRKNA   180
MB1569_ST2         YKAANNKIYPTINDGEQVVANGVYDTETKKLVYTFTDYVNKKDNIKGQFEIPQFIDRKNA   180
MB1985_ST2         YKAANNKIYPTINDGEQVVANGVYDTETKKLVYTFTDYVNKKDNIKGQFEIPQFIDRKNA   180
                   ************************************************************

MB905_ST5          KTSGDYDLNYNIADKTVSKPMKIVYNNYDEGHVVANTSSLITKADLFNVGSHDYTQYIYV   240
MB1143_ST210       KTSGDYDLNYNIADKTVSKPMKIVYNNYDEGHVVANTSSLITKADLFNVGSHDYTQYIYV   240
MB1312_St5         KTSGDYDLNYNIADKTVSKPMKIVYNNYDEGHVVANTSSLITKADLFNVGSHDYTQYIYV   240
MB1709_ST210       KTSGDYDLNYNIADKTVSKPMKIVYNNYDEGHVVANTSSLITKADLFNVGSHDYTQYIYV   240
MB1715_ST210       KTSGDYDLNYNIADKTVSKPMKIVYNNYDEGHVVANTSSLITKADLFNVGSHDYTQYIYV   240
MB1907_ST210       KTSGDYDLNYNIADKTVSKPMKIVYNNYDEGHVVANTSSLITKADLFNVGSHDYTQYIYV   240
MB526_ST210        KTSGDYDLNYNIADKTVSKPMKIVYNNYDEGHVVANTSSLITKADLFNVGSHDYTQYIYV   240
MB896_ST5          KTSGDYDLNYNIADKTVSKPMKIVYNNYDEGHVVANTSSLITKADLFNVGSHDYTQYIYV   240
MB2193_ST5         KTSGDYDLNYNIADKTVSKPMKIVYNNYDEGHVVANTSSLITKADLFNVGSHDYTQYIYV   240
MB740_ST5          KTSGDYDLNYNIADKTVSKPMKIVYNNYDEGHVVANTSSLITKADLFNVGSHDYTQYIYV   240
MB972_ST5          KTSGDYDLNYNIADKTVSKPMKIVYNNYDEGHVVANTSSLITKADLFNVGSHDYTQYIYV   240
MB1037_ST5         KTSGDYDLNYNIADKTVSKPMKIVYNNYDEGHVVANTSSLITKADLFNVGSHDYTQYIYV   240
MB1064_ST5         KTSGDYDLNYNIADKTVSKPMKIVYNNYDEGHVVANTSSLITKADLFNVGSHDYTQYIYV   240
MB1489_ST5         KTSGDYDLNYNIADKTVSKPMKIVYNNYDEGHVVANTSSLITKADLFNVGSHDYTQYIYV   240
MB1586_ST210       KTSGDYDLNYNIADKTVSKPMKIVYNNYDEGHVVANTSSLITKADLFNVGSHDYTQYIYV   240
MB1679_ST5         KTSGDYDLNYNIADKTVSKPMKIVYNNYDEGHVVANTSSLITKADLFNVGSHDYTQYIYV   240
MB1691a_ST5        KTSGDYDLNYNIADKTVSKPMKIVYNNYDEGHVVANTSSLITKADLFNVGSHDYTQYIYV   240
MB988_ST5          KTSGDYDLNYNIADKTVSKPMKIVYNNYDEGHVVANTSSLITKADLFNVGSHDYTQYIYV   240
MB1695_ST5         KTSGDYDLNYNIADKTVSKPMKIVYNNYDEGHVVANTSSLITKADLFNVGSHDYTQYIYV   240
MB1961_ST5         KTSGDYDLNYNIADKTVSKPMKIVYNNYDEGHVVANTSSLITKADLFNVGSHDYTQYIYV   240
MB383_ST5          KTSGDYDLNYNIADKTVSKPMKIVYNNYDEGHVVANTSSLITKADLFNVGSHDYTQYIYV   240
MB10_ST5           KTSGDYDLNYNIADKTVSKPMKIVYNNYDEGHVVANTSSLITKADLFNVGSHDYTQYIYV   240
MB1048_ST2         KTSGDYDLNYNIADKTVSKPMKIVYNNYDEGHVVANTSSLITKADLFNVGSHDYTQYIYV   240
MB1299_ST57        KTSGDYDLNYNIADKTVSKPMKIVYNNYDEGHVVANTSSLITKADLFNVGSHDYTQYIYV   240
MB1306_ST2         KTSGDYDLNYNIADKTVSKPMKIVYNNYDEGHVVANTSSLITKADLFNVGSHDYTQYIYV   240
MB1651_ST2         KTSGDYDLNYNIADKTVSKPMKIVYNNYDEGHVVANTSSLITKADLFNVGSHDYTQYIYV   240
MB1750_ST2         KTSGDYDLNYNIADKTVSKPMKIVYNNYDEGHVVANTSSLITKADLFNVGSHDYTQYIYV   240
MB567_ST218        KTSGDYDLNYNIADKTVSKPMKIVYNNYDEGHVVANTSSLITKADLFNVGSHDYTQYIYV   240
MB1569_ST2         KTSGDYDLNYNIADKTVSKPMKIVYNNYDEGHVVANTSSLITKADLFNVGSHDYTQYIYV   240
MB1985_ST2         KTSGDYDLNYNIADKTVSKPMKIVYNNYDEGHVVANTSSLITKADLFNVGSHDYTQYIYV   240
                   ************************************************************

MB905_ST5          NPKSEDSYNTRLTIQGYQEDLNNSSTLLNPKDSNIEILDAKSSDNITPSFYVNDSDFENV   300
MB1143_ST210       NPKSEDSYNTRLTIQGYQEDLNNSSTLLNPKDSNIEILDAKSSDNITPSFYVNDSDFENV   300
MB1312_St5         NPKSEDSYNTRLTIQGYQEDLNNSSTLLNPKDSNIEILDAKSSDNITPSFYVNDSDFENV   300
```

```
MB1709_ST210      NPKSEDSYNTRLTIQGYQEDLNNSSTLLNPKDSNIEILDAKSSDNITPSFYVNDSDFENV   300
MB1715_ST210      NPKSEDSYNTRLTIQGYQEDLNNSSTLLNPKDSNIEILDAKSSDNITPSFYVNDSDFENV   300
MB1907_ST210      NPKSEDSYNTRLTIQGYQEDLNNSSTLLNPKDSNIEILDAKSSDNITPSFYVNDSDFENV   300
MB526_ST210       NPKSEDSYNTRLTIQGYQEDLNNSSTLLNPKDSNIEILDAKSSDNITPSFYVNDSDFENV   300
MB896_ST5         NPKSEDSYNTRLTIQGYQEDLNNSSTLLNPKDSNIEILDAKSSDNITPSFYVNDSDFENV   300
MB2193_ST5        NPKSEDSYNTRLTIQGYQEDLNNSSTLLNPKDSNIEILDAKSSDNITPSFYVNDSDFENV   300
MB740_ST5         NPKSEDSYNTRLTIQGYQEDLNNSSTLLNPKDSNIEILDAKSSDNITPSFYVNDSDFENV   300
MB972_ST5         NPKSEDSYNTRLTIQGYQEDLNNSSTLLNPKDSNIEILDAKSSDNITPSFYVNDSDFENV   300
MB1037_ST5        NPKSEDSYNTRLTIQGYQEDLNNSSTLLNPKDSNIEILDAKSSDNITPSFYVNDSDFENV   300
MB1064_ST5        NPKSEDSYNTRLTIQGYQEDLNNSSTLLNPKDSNIEILDAKSSDNITPSFYVNDSDFENV   300
MB1489_ST5        NPKSEDSYNTRLTIQGYQEDLNNSSTLLNPKDSNIEILDAKSSDNITPSFYVNDSDFENV   300
MB1586_ST210      NPKSEDSYNTRLTIQGYQEDLNNSSTLLNPKDSNIEILDAKSSDNITPSFYVNDSDFENV   300
MB1679_ST5        NPKSEDSYNTRLTIQGYQEDLNNSSTLLNPKDSNIEILDAKSSDNITPSFYVNDSDFENV   300
MB1691a_ST5       NPKSEDSYNTRLTIQGYQEDLNNSSTLLNPKDSNIEILDAKSSDNITPSFYVNDSDFENV   300
MB988_ST5         NPKSEDSYNTRLTIQGYQEDLNNSSTLLNPKDSNIEILDAKSSDNITPSFYVNDSDFENV   300
MB1695_ST5        NPKSEDSYNTRLTIQGYQEDLNNSSTLLNPKDSNIEILDAKSSDNITPSFYVNDSDFENV   300
MB1961_ST5        NPKSEDSYNTRLTIQGYQEDLNNSSTLLNPKDSNIEILDAKSSDNITPSFYVNDSDFENV   300
MB383_ST5         NPKSEDSYNTRLTIQGYQEDLNNSSTLLNPKDSNIEILDAKSSDNITPSFYVNDSDFENV   300
MB10_ST5          NPKSEDSYNTRLTIQGYQEDLNNSSTLLNPKDSNIEILDAKSSDNITPSFYVNDSDFENV   300
MB1048_ST2        NPKSEDSYNTRLTIQGYQEDVNDSSTLLNPDDTKIEILDAKSSDNIVPSFHINDEDFEDV   300
MB1299_ST57       NPKSEDSYNTRLTIQGYQEDVNDSSTLLNPDDTKIEILDAKSSDNIVPSFHINDEDFEDV   300
MB1306_ST2        NPKSEDSYNTRLTIQGYQEDVNDSSTLLNPDDTKIEILDAKSSDNIVPSFHINDEDFEDV   300
MB1651_ST2        NPKSEDSYNTRLTIQGYQEDVNDSSTLLNPDDTKIEILDAKSSDNIVPSFHINDEDFEDV   300
MB1750_ST2        NPKSEDSYNTRLTIQGYQEDVNDSSTLLNPDDTKIEILDAKSSDNIVPSFHINDEDFEDV   300
MB567_ST218       NPKSEDSYNTRLTIQGYQEDVNDSSTLLNPDDTKIEILDAKSSDNIVPSFHINDEDFEDV   300
MB1569_ST2        NPKSEDSYNTRLTIQGYQEDVNDSSTLLNPDDTKIEILDAKSSDNIVPSFHINDEDFEDV   300
MB1985_ST2        NPKSEDSYNTRLTIQGYQEDVNDSSTLLNPDDTKIEILDAKSSDNIVPSFHINDEDFEDV   300
                  ********************:*:*******.*::************.***::**.***:*

MB905_ST5         TNQYKIDQIGDKKAQIDFGHIDHPYIVKVTSKIDPNSSKDLRTRVIMENENAEGTKDFYV   360
MB1143_ST210      TNQYKIDQIGDKKAQIDFGHIDHPYIVKVTSKIDPNSSKDLRTRVIMENENAEGTKDFYV   360
MB1312_St5        TNQYKIDQIGDKKAQIDFGHIDHPYIVKVTSKIDPNSSKDLRTRVIMENENAEGTKDFYV   360
MB1709_ST210      TNQYKIDQIGDKKAQIDFGHIDHPYIVKVTSKIDPNSSKDLRTRVIMENENAEGTKDFYV   360
MB1715_ST210      TNQYKIDQIGDKKAQIDFGHIDHPYIVKVTSKIDPNSSKDLRTRVIMENENAEGTKDFYV   360
MB1907_ST210      TNQYKIDQIGDKKAQIDFGHIDHPYIVKVTSKIDPNSSKDLRTRVIMENENAEGTKDFYV   360
MB526_ST210       TNQYKIDQIGDKKAQIDFGHIDHPYIVKVTSKIDPNSSKDLRTRVIMENENAEGTKDFYV   360
MB896_ST5         TNQYKIDQIGDKKAQIDFGHIDHPYIVKVTSKIDPNSSKDLRTRVIMENENAEGTKDFYV   360
MB2193_ST5        TNQYKIDQIGDKKAQIDFGHIDHPYIVKVTSKIDPNSSKDLRTRVIMENENAEGTKDFYV   360
MB740_ST5         TNQYKIDQIGDKKAQIDFGHIDHPYIVKVTSKIDPNSSKDLRTRVIMENENAEGTKDFYV   360
MB972_ST5         TNQYKIDQIGDKKAQIDFGHIDHPYIVKVTSKIDPNSSKDLRTRVIMENENAEGTKDFYV   360
MB1037_ST5        TNQYKIDQIGDKKAQIDFGHIDHPYIVKVTSKIDPNSSKDLRTRVIMENENAEGTKDFYV   360
MB1064_ST5        TNQYKIDQIGDKKAQIDFGHIDHPYIVKVTSKIDPNSSKDLRTRVIMENENAEGTKDFYV   360
MB1489_ST5        TNQYKIDQIGDKKAQIDFGHIDHPYIVKVTSKIDPNSSKDLRTRVIMENENAEGTKDFYV   360
MB1586_ST210      TNQYKIDQIGDKKAQIDFGHIDHPYIVKVTSKIDPNSSKDLRTRVIMENENAEGTKDFYV   360
MB1679_ST5        TNQYKIDQIGDKKAQIDFGHIDHPYIVKVTSKIDPNSSKDLRTRVIMENENAEGTKDFYV   360
MB1691a_ST5       TNQYKIDQIGDKKAQIDFGHIDHPYIVKVTSKIDPNSSKDLRTRVIMENENAEGTKDFYV   360
MB988_ST5         TNQYKIDQIGDKKAQIDFGHIDHPYIVKVTSKIDPNSSKDLRTRVIMENENAEGTKDFYV   360
MB1695_ST5        TNQYKIDQIGDKKAQIDFGHIDHPYIVKVTSKIDPNSSKDLRTRVIMENENAEGTKDFYV   360
MB1961_ST5        TNQYKIDQIGDKKAQIDFGHIDHPYIVKVTSKIDPNSSKDLRTRVIMENENAEGTKDFYV   360
MB383_ST5         TNQYKIDQIGDKKAQIDFGHIDHPYIVKVTSKIDPNSSKDLRTRVIMENENAEGTKDFYV   360
MB10_ST5          TNQYKIDQIGDKKAQIDFGHIDHPYIVKVTSKIDPNSSKDLRTRVIMENENAEGTKDFYV   360
MB1048_ST2        TGNFGINQKGDKKAQIDFGHIDHPYIVKVTSKIDPSSSQDLRTRVIMENENAEGTTDFYA   360
MB1299_ST57       TGNFGINQKGDKKAQIDFGHIDHPYIVKVTSKIDPSSSQDLRTRVIMENENAEGTTDFYA   360
MB1306_ST2        TGNFGINQKGDKKAQIDFGHIDHPYIVKVTSKIDPSSSQDLRTRVIMENENAEGTTDFYA   360
MB1651_ST2        TGNFGINQKGDKKAQIDFGHIDHPYIVKVTSKIDPSSSQDLRTRVIMENENAEGTTDFYA   360
MB1750_ST2        TGNFGINQKGDKKAQIDFGHIDHPYIVKVTSKIDPSSSQDLRTRVIMENENAEGTTDFYA   360
MB567_ST218       TGNFGINQKGDKKAQIDFGHIDHPYIVKVTSKIDPSSSQDLRTRVIMENENAEGTTDFYA   360
MB1569_ST2        TGNFGINQKGDKKAQIDFGHIDHPYIVKVTSKIDPSSSQDLRTRVIMENENAEGTTDFYA   360
MB1985_ST2        TGNFGINQKGDKKAQIDFGHIDHPYIVKVTSKIDPSSSQDLRTRVIMENENAEGTTDFYA   360
                  *.:: *:* **************************.**:****************.***.

MB905_ST5         HDNTVERLGANGVATGNEK        379
MB1143_ST210      HDNTVERLGANGVATGNEK        379
MB1312_St5        HDNTVERLGANGVATGNEK        379
MB1709_ST210      HDNTVERLGANGVATGNEK        379
MB1715_ST210      HDNTVERLGANGVATGNEK        379
MB1907_ST210      HDNTVERLGANGVATGNEK        379
```

```
MB526_ST210       HDNTVERLGANGVATGNEK       379
MB896_ST5         HDNTVERLGANGVATGNEK       379
MB2193_ST5        HDNTVERLGANGVATGNEK       379
MB740_ST5         HDNTVERLGANGVATGNEK       379
MB972_ST5         HDNTVERLGANGVATGNEK       379
MB1037_ST5        HDNTVERLGANGVATGNEK       379
MB1064_ST5        HDNTVERLGANGVATGNEK       379
MB1489_ST5        HDNTVERLGANGVATGNEK       379
MB1586_ST210      HDNTVERLGANGVATGNEK       379
MB1679_ST5        HDNTVERLGANGVATGNEK       379
MB1691a_ST5       HDNTVERLGANGVATGNEK       379
MB988_ST5         HDNTVERLGANGVATGNEK       379
MB1695_ST5        HDNTVERLGANGVATGNEK       379
MB1961_ST5        HDNTVERLGANGVATGNEK       379
MB383_ST5         HDNTVERLGANGVATGNEK       379
MB10_ST5          HDNTVERLGANGVATGNEK       379
MB1048_ST2        HDNTVERLGANGVATGNEK       379
MB1299_ST57       HDNTVERLGANGVATGNEK       379
MB1306_ST2        HDNTVERLGANGVATGNEK       379
MB1651_ST2        HDNTVERLGANGVATGNEK       379
MB1750_ST2        HDNTVERLGANGVATGNEK       379
MB567_ST218       HDNTVERLGANGVATGNEK       379
MB1569_ST2        HDNTVERLGANGVATGNEK       379
MB1985_ST2        HDNTVERLGANGVATGNEK       379
                  *******************
```
