# Supplementary material for: Staphylococcus epidermidis MSCRAMM SesJ Is Encoded in Composite Islands
Source: mBio. 2020 Feb 18;11(1):e02911-19. doi: 10.1128/mBio.02911-19 (PMC7029136; doi:10.1128/mBio.02911-19)
Supplement: TABLE S1 [file mBio.02911-19-st001.pdf]

Table S1. Primer used for confirming MGE structures

Primer name	Primer sequence
1F	TACAAACACGCATGACAC
1F-2	AATAAGATTAAACCCCAATCTACAG
1R	GCTAGCTACAATGCCAAA
1R-2	ACTTCACATCTATTAGGTTATGTTG
2F	CATTTCTACTTCACCATTATCG
2R-2	GAAGCGACCAGTTGTTATC
3F	GATGGAAGTCACAGTATTCTTTG
3R-2	CAATTGTTCTTCATTTATAAATGG
4F-2	ACTGTATTAGTTTAATCAGAAGACGTG
5F	TCGAGGGTGTAGAAGTAT
5R	CGTTGAAGTAAAGAGGTC
6F	ATCGTAATAGAGTGGTGG
6F-2	CGTAATAGAGTGGTGGCTTCAGTAG
6R	GAAAGAATAGGAAGTGGAAG
6R-2	TTATGGGGCAATCTCCTAAAAGTGA
7F	GTCGTAGCCTAGTGATTGTAGC
7R-2	GGATGGGAGACTTGACTACTG
8F	GATTGGCCAAGTGATATTC
8R-2	CGCTTTAGACGGTAAAATAG
9F	CTTGTAAGTCACGAGAAATAGTTG
9R-2	GTGCTCATTTTTAACAACGAC
10F	GATGCAGGTTGGAAGTAAAC
10R-2	GAGCCTTTTGATAAACATACAG
11F-2	CAGTACATATGAAAAGTATGGTGAT
11R-3	CTTTTGAGAGTTCTTTGTAATGATG
12F	GGACTATGCCAATATAGAAAATC
12R-2	CATATAAGCATCTTCAAAATCAG
13F	TAGACGGTAAGTCAGTGAG
13F-2	TCTCATTAGACGGTAAGTCAGTGAG
13R	GAGGCTTATCATGGTGGA
13R-2	GGCTTATCATGGTGGACATATTGAAG
14R	AACTACATGTCCCTCATC
14R-2	TCCCTCATCGTAGTTGTTGTATAC
15F	AGTGTGAGATGAGTAAGG
16F	TGGACTCATCACTGACTA
16R	TCCATCTTCTCCAACAGC
17F	ACCAGCCTCCTATTACTTA
17R	CCATCCCTCTTGATTCCA
18R	ATTCCCCTTACCTTTACC
19F	CATATTCTGCTAAACCTTCG

20F	CCTGACACGTATTCTTCT
21R	CTGATAAGCGGAATGGTT
22F	TTAGGGTTGAGTGGATG
24F	TTAGAGCATTTAGGGGTGTT
24R	TTAGGGGCTTGTTCGGTT
25R	TGAGGTTATTCAGATATTTCGATG
26F	ACATGGAGAATCCGTGATTCATTTG
26R	GGAGATACTACTGCTTCATCGACTT
CcrC2F	CCATTACAACCGTACTTAATC
CcrC2R	GGTCGATGAAATAATCATTTTG
CcrC8F	CAATCACGACAATTCTAAATC
CcrC8R	CAATCCTATTTTCATTTGTGAC
