# Supplementary material for: Staphylococcus epidermidis MSCRAMM SesJ Is Encoded in Composite Islands
Source: mBio. 2020 Feb 18;11(1):e02911-19. doi: 10.1128/mBio.02911-19 (PMC7029136; doi:10.1128/mBio.02911-19)
Supplement: TABLE S2 [file mBio.02911-19-st002.pdf]

Table S2: Amino acid identity comparison of SesJ associated GtfA and GtfB with other staphylococcal glycosyltransferases encoded in the core genome.

Host	*S. epidermidis*	*S. epidermidis*	*S. epidermidis*	*S. epidermidis*	*S. aureus*	*S. aureus*
Accession No			AAO06036.1	AAO03929.1	BAF66798.1	BAF66799.1
Glycosyl-transferase	GtfA	GtfB	SdrF_Gtf	SdrG_Gtf	SdgA	SdgB
GtfA	100	17.88	22.55	24.47	24.68	22.01
GtfB	17.88	100	16.52	15.83	15.52	18.14
SdrF_Gtf	22.55	16.62	100	46.06	41.98	47.36
SdrG_Gtf	24.27	15.83	46.06	100	44.08	54.84
SdgA	24.68	15.52	41.98	44.08	100	43.47
SdgB	22.01	18.14	47.36	54.84	43.47	100
